# Anti-Allergic and Anti-Inflammatory Effects of Undecane on Mast Cells and Keratinocytes

**DOI:** 10.3390/molecules25071554

**Published:** 2020-03-28

**Authors:** Dabin Choi, Wesuk Kang, Taesun Park

**Affiliations:** Department of Food and Nutrition, Brain Korea 21 PLUS Project, Yonsei University, 50 Yonsei-ro, Seodaemun-gu, Seoul 03722, Korea; vin1411@naver.com (D.C.); wesuk42@naver.com (W.K.)

**Keywords:** undecane, inflammation, allergy, histamine, cAMP

## Abstract

The critical roles of keratinocytes and resident mast cells in skin allergy and inflammation have been highlighted in many studies. Cyclic adenosine monophosphate (cAMP), the intracellular second messenger, has also recently emerged as a target molecule in the immune reaction underlying inflammatory skin conditions. Here, we investigated whether undecane, a naturally occurring plant compound, has anti-allergic and anti-inflammatory activities on sensitized rat basophilic leukemia (RBL-2H3) mast cells and HaCaT keratinocytes and we further explored the potential involvement of the cAMP as a molecular target for undecane. We confirmed that undecane increased intracellular cAMP levels in mast cells and keratinocytes. In sensitized mast cells, undecane inhibited degranulation and the secretion of histamine and tumor necrosis factor α (TNF-α). In addition, in sensitized keratinocytes, undecane reversed the increased levels of p38 phosphorylation, nuclear factor kappaB (NF-κB) transcriptional activity and target cytokine/chemokine genes, including thymus and activation-regulated chemokine (TARC), macrophage-derived chemokine (MDC) and interleukin-8 (IL-8). These results suggest that undecane may be useful for the prevention or treatment of skin inflammatory disorders, such as atopic dermatitis, and other allergic diseases.

## 1. Introduction

Mast cells are hematopoietic cells derived in the bone marrow from progenitor cells. They are involved in the pathophysiology of allergic diseases, especially in IgE-mediated hypersensitivity reactions in the skin and gastrointestinal tract, such as atopic dermatitis (AD) and asthma [1,2]. Mast cells express the high-affinity IgE receptor, FcεRI, on their surface and can be stimulated by antigen-regulated aggregation of IgE bound to the receptor. Antigen then leads to degranulation of mast cells, secreting a broad array of mediators, such as cytokines/chemokines and biogenic amines including tumor necrosis factor α (TNF-α) and histamine [3,4]. It is also noteworthy that the presence of these mediators plays an important role in regulating inflammatory responses in other immune-related cells such as keratinocytes and lymphocytes [5]. In particular, activated keratinocytes release a variety of proinflammatory cytokines and chemokines that can sustain allergic reactions, thus possibly contributing to AD [6].

Cyclic adenosine monophosphate (cAMP), identified as the major second messenger for extracellular ligand action in 1968, is now accepted as a master regulator in diverse physiological processes [7]. In particular, this second messenger has recently emerged as an important player in the immune system underlying inflammatory skin diseases such as AD. Modulations of intracellular cAMP, thus, can greatly affect immune responses in a variety of immune cells in a cell-specific manner, where increased cAMP generally suppresses inflammatory response [8]. A large number of studies over more than five decades have revealed that cAMP has a critical role in inhibiting a pathologically increased mast cell activity by reducing calcium flux, which is critically required for mast cell degranulation. For example, elevations in the intracellular levels of cAMP induced by diverse stimuli, including adenosine and β-adrenoceptor agonists, have been shown to dampen mast cell responses [9,10,11]. On the other hand, in keratinocytes, cAMP-induced protein kinase A (PKA) activation results in cAMP-response element-binding protein (CREB) phosphorylation, resulting an the increase of anti-inflammatory cytokines [12]. It has also been reported that PKA activation decreases the transcriptional activity of nuclear factor kappaB (NF-κB), one of the master regulators of inflammation, possibly via p38 activation [12,13,14].

Steroids and immunosuppressants currently remain the most common treatment for most allergic inflammatory skin disorders such as AD. However, conventional treatments have shown limited improved efficacy because of the development of pharmacoresistance and accompanying side effects [15,16,17,18]. Using phytochemical library screening in mast cells, we identified that undecane inhibited inflammatory mediators such as histamine. Undecane is a naturally occurring plant compound mainly found in *Salvia verbenaca* seed oil and *Dialium guineense* fruit oil [19]; however, its biological activity is still unknown. The goal of this investigation was to evaluate the anti-allergic and anti-inflammatory activities of undecane on sensitized rat basophilic leukemia (RBL-2H3) mast cells and HaCaT keratinocytes and to explore the potential involvement of the cAMP as a molecular target for undecane in the process of reducing immune responses.

## 2. Results

### 2.1. Undecane Has no Effect on Cell Viability in RBL-2H3 Cells and HaCaT Cells

**Figure 1:**
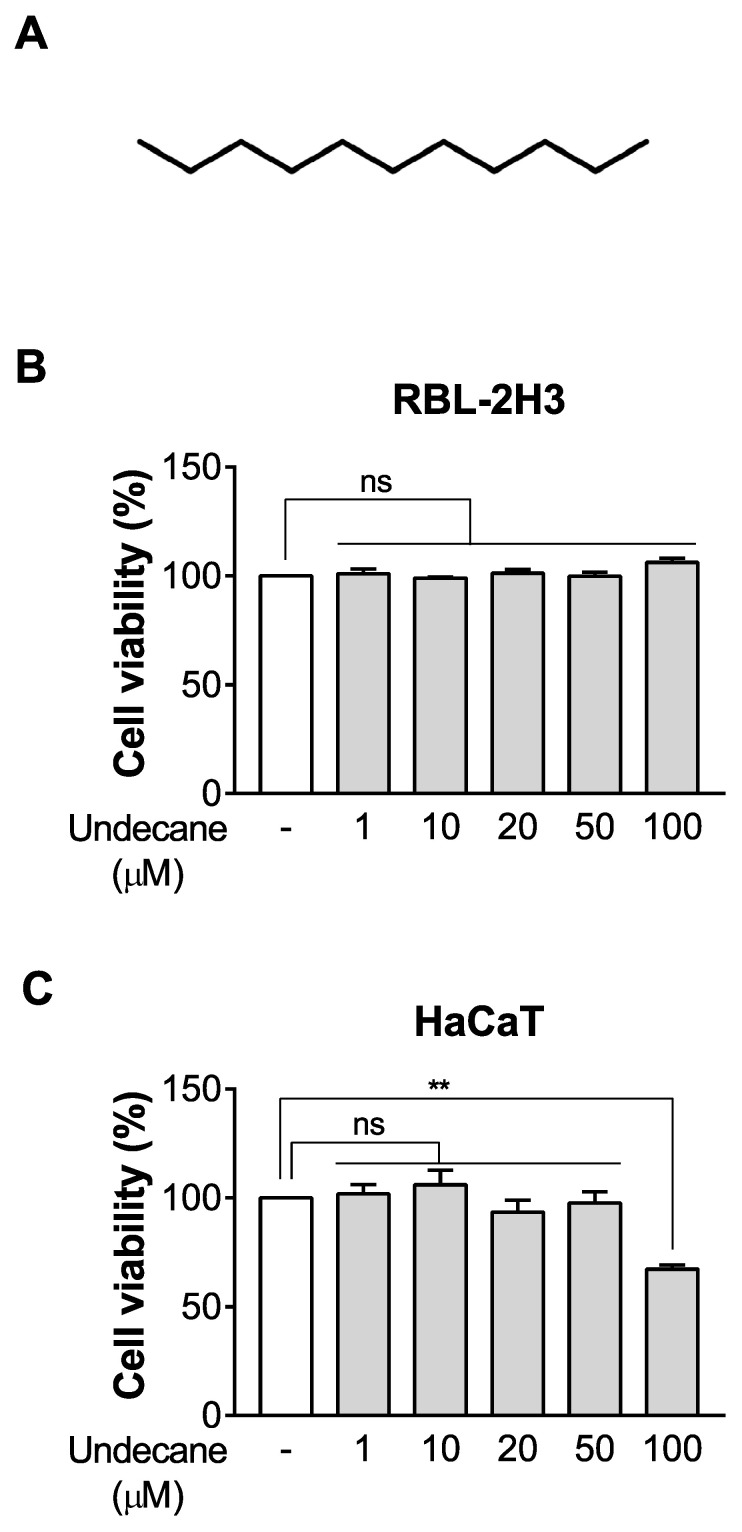
Effect of undecane on cell viability in RBL-2H3 cells and HaCaT cells. (**A**) The structure of undecane. (**B**) Cell viability was determined after incubation with vehicle (DMSO) or various concentrations of undecane (1, 10, 20, 50 and 100 μM) in RBL-2H3 cells and (**C**) HaCaT cells for 24 h. The data were expressed as the mean ± SEM (*n* = 3). Significant differences between groups are indicated by asterisks; ns, not significant (*p* > 0.05); ** *p* < 0.01.

The structure of undecane is shown in Figure 1A. The 3-[4-C-dimethylthiazole-2-yl]-2,5-diphenyltetrazolium bromide (MTT) assay showed that undecane (1–100 μM) did not affect the cell viability of RBL-2H3 cells (Figure 1B). We also found that undecane had no effect on the cell viability of HaCaT cells at all concentrations except 100 μM compared to the control group (Figure 1C).

### 2.2. Undecane Suppresses Degranulation of Sensitized RBL-2H3 Cells

**Figure 2:**
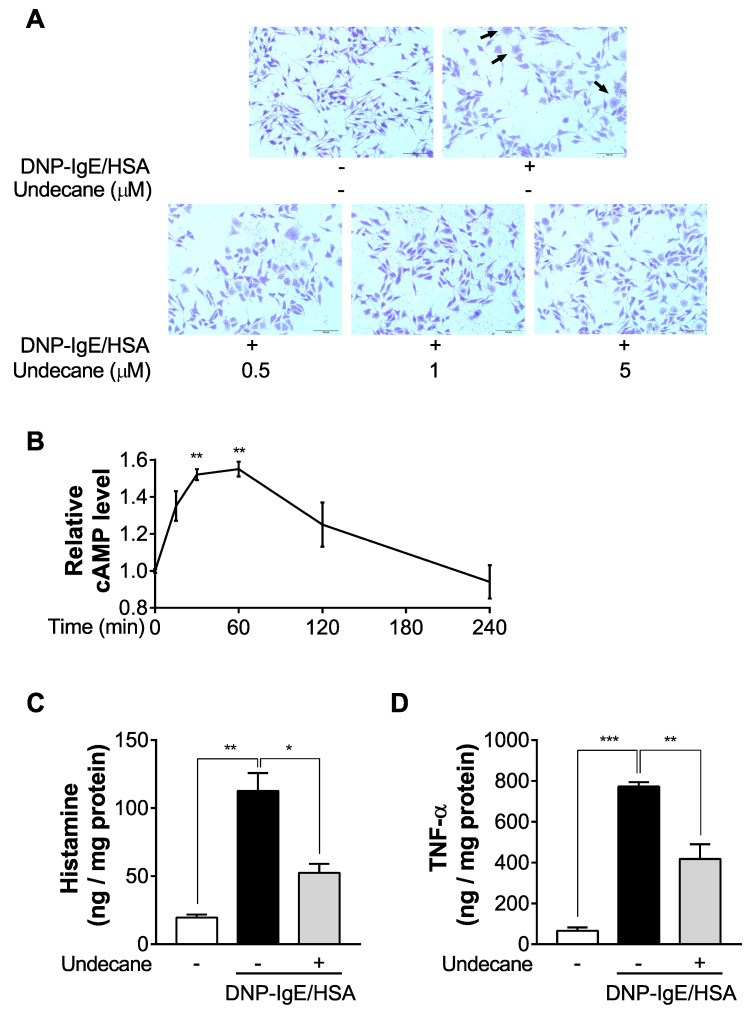
Effect of undecane on degranulation in dinitrophenyl immunoglobulin E/human serum albumin (DNP-IgE/HAS) stimulated RBL-2H3 cells. RBL-2H3 cells were pretreated with gradient concentrations of undecane (0.5, 1 and 5 μM) for 1 h and stimulated with DNP-IgE/HSA (50 ng/mL each) for 5 h. After that, (**A**) the images of toluidine blue-stained cells were captured. Arrows show that the cell morphology was irregular and unclear. Purple granules were secreted outside the cells. The scale bar represents 100 μm. (**B**) RBL-2H3 cells were incubated with undecane (1 μM) for various times and the effect of undecane on intracellular cAMP levels was determined. RBL-2H3 cells were preincubated with undecane for 1 h and stimulated with DNP-IgE/HSA (50 ng/mL each) for 5 h. (**C**) The histamine and (**D**) tumor necrosis factor α (TNF-α) concentrations were then measured in the supernatants. The final concentrations were normalized to the total cellular protein amount. The data were expressed as the mean ± SEM (*n* = 3). Significant differences between groups are indicated by asterisks; * *p* < 0.05; ** *p* < 0.01; *** *p* < 0.001.

To evaluate whether undecane treatment inhibits dinitrophenyl immunoglobulin E/human serum albumin (DNP-IgE/HSA)-stimulated degranulation in mast cells, toluidine blue staining and histamine/TNF-α release assay were performed. While we confirmed an elongated shape structure and dense intracellular granule content in the control group, DNP-HSA stimulation resulted in typical degranulated morphology characterized by irregular shapes and reduced granule content as evidenced by staining intensities. Notably, undecane significantly inhibited the DNP-IgE/HSA-induced morphological changes and degranulation in a dose-dependent manner (Figure 2A). We further measured cAMP concentration to examine whether the inhibitory effect of undecane on cell degranulation was related to intracellular cAMP. We confirmed that undecane (1 μM) significantly increased the levels of intracellular cAMP in RBL-2H3 mast cells. The peak elevation of cAMP levels was seen at 30–60 min and the intracellular cAMP concentration returned to the basal value by 240 min (Figure 2B). The release of histamine and TNF-α was markedly upregulated in the DNP-IgE/HSA group compared with the basal group, but undecane significantly inhibited the histamine and TNF-α release compared with DNP-IgE/HSA control (Figure 2C,D).

### 2.3. Undecane Regulates cAMP-Mediated Inflammatory Signaling Pathway in HaCaT Cells

**Figure 3:**
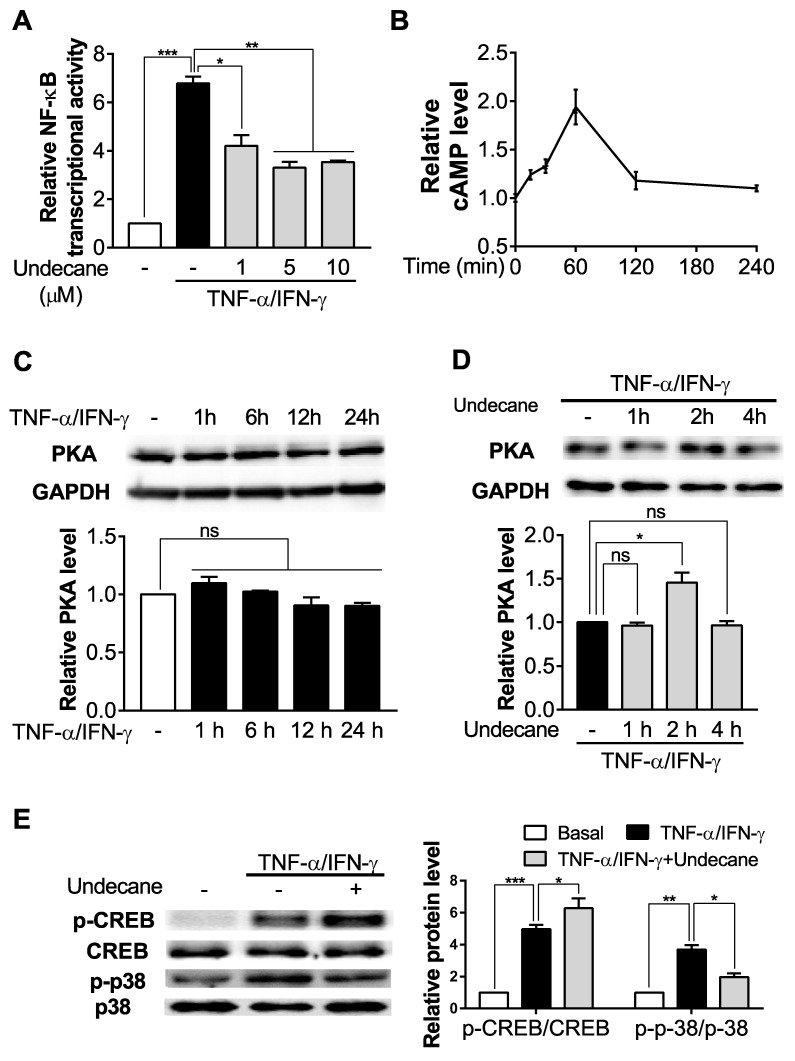
Effect of undecane on cyclic adenosine monophosphate (cAMP)-mediated inflammatory signaling pathway in HaCaT cells. (**A**) After pretreatment (1 h) of vehicle (DMSO) or undecane (1, 5 and 10 μM), the cells were incubated with TNF-α/IFN-γ (10 ng/mL each) for 1 h and nuclear factor kappaB (NF-κB) transcriptional activity was measured in nuclear extraction of each group. (**B**) HaCaT cells were incubated with undecane (5 μM) for various times and the effect of undecane on intracellular cAMP levels was determined. Western blot was performed to assess the protein levels of (**C**, **D**) protein kinase A (PKA), (**E**) p-CREB, cAMP-response element-binding protein (CREB), p-p38 and p38 in HaCaT cells. (**C**) HaCaT cells were incubated with TNF-α/IFN-γ (10 ng/mL each) for various times and PKA level was measured. (**D**) In a similar manner, TNF-α/IFN-γ-stimulated HaCaT cells were cultured with vehicle or undecane for various times and PKA level was determined. (**E**) After pretreatment (1 h) of vehicle or undecane (5 μM), the cells were incubated with TNF-α/IFN-γ for 1 h and phosphorylation of CREB and p38 was measured. All the protein expression data were normalized against glyceraldehyde 3-phosphate dehydrogenase (GAPDH) level. The data were expressed as the mean ± SEM (*n* = 3). Significant differences between groups are indicated by asterisks; ns, not significant (*p* > 0.05); * *p* < 0.05; ** *p* < 0.01; *** *p* < 0.001.

To find whether undecane has anti-allergic and anti-inflammatory activities in HaCaT cells and if these effects were regulated by the cAMP signaling pathway, we first assessed the NF-κB p65 DNA-binding activity. TNF-α/interferon gamma (IFN-γ) treatment markedly increased the NF-κB transcriptional activity in nucleus compared to controls, but treatment with gradient concentrations of undecane (1, 5 and 10 μM) significantly inhibited increased NF-κB activation in TNF-α/IFN-γ-stimulated HaCaT cells in a dose-dependent manner, which was saturated around 5 μM (Figure 3A). Thereafter, we evaluated intracellular cAMP levels affected by undecane treatment (5 μM). The peak elevation of cAMP levels was seen at 60 min and the concentration was returned to the basal value by 120 min (Figure 3B). Furthermore, our data also showed that TNF-α/IFN-γ treatment did not significantly change the protein level of PKA (Figure 3C) while undecane enhanced the protein levels of PKA compared with basal in 2 h (Figure 3D). TNF-α/IFN-γ treatment significantly increased the phosphorylation of both CREB and p38. Treatment of undecane significantly enhanced phospho-CREB (p-CREB) and reversed the upregulation of p-p38 (Figure 3E).

### 2.4. Undecane Reduces the mRNA Expression of Inflammatory Cytokines in HaCaT Cells

**Figure 4:**
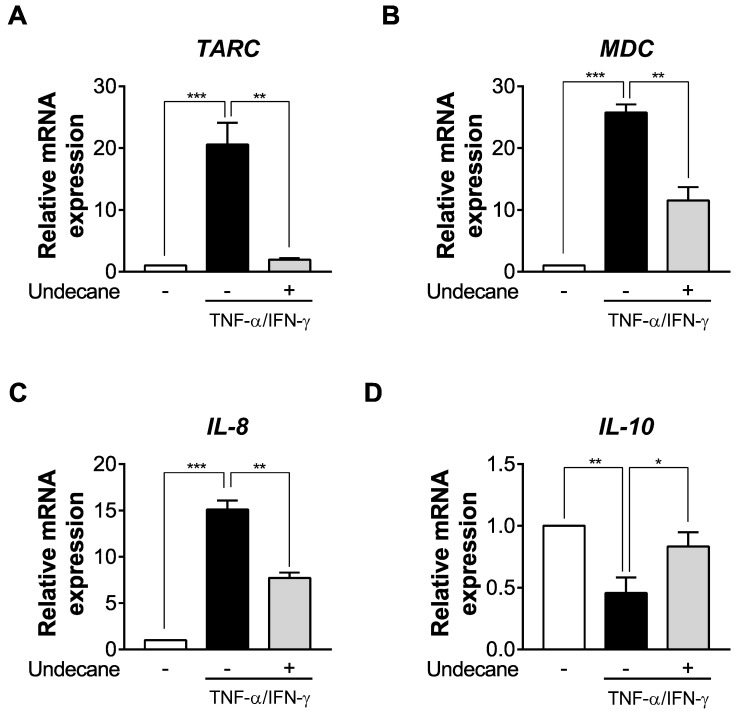
Effect of undecane on mRNA expression of inflammatory cytokines in HaCaT cells. After pretreatment (1 h) of vehicle (DMSO) or undecane (5 μM), the cells were incubated with TNF-α/IFN-γ for 6 h and the mRNA expression levels of the proinflammatory cytokines such as (**A**) thymus and activation-regulated chemokine (TARC), (**B**) macrophage-derived chemokine (MDC) and (**C**) interleukin-8 (IL-8) and anti-inflammatory cytokine, (**D**) interleukin-10 (IL-10), were determined. All gene expression data were normalized against GAPDH. The data were expressed as the mean ± SEM (*n* = 3). Significant differences between groups are indicated by asterisks; * *p* < 0.05; ** *p* < 0.01; *** *p* < 0.001.

To investigate whether undecane attenuated the TNF-α/IFN-γ-mediated inflammation in HaCaT cells, we evaluated the gene expression of NF-κB related inflammatory cytokines. In parallel to NF-κB activity results, the mRNA expression of proinflammatory cytokines such as thymus and activation-regulated chemokine (TARC), macrophage-derived chemokine (MDC) and interleukin-8 (IL-8) (Figure 4A–C) were dramatically increased in the TNF-α/IFN-γ treatment group compared with the control group, while undecane significantly reduced mRNA expression of these cytokines. In addition, undecane reversed the TNF-α /IFN-γ-induced reduction of IL-10 mRNA levels (Figure 4D).

## 3. Discussion

Although it has been reported that various proinflammatory molecules are associated with an allergic reaction, histamine is still the major inflammatory regulator that is responsible for, at least, half of the symptoms and signs of the allergic response in the skin [20]. Functional diversity of histamine as a mediator of allergic symptoms can be derived from a variety of cell types that express different histamine receptors (H1-4 receptors) [21,22]. For example, it has been accepted that typical immediate hypersensitivity responses of skin allergic reactions, such as swelling, redness and itching, are the result of histamine H1 receptor activation in epithelial and nerve cells [23,24]. In addition, Schmelz et al. demonstrated that histamine delayed the barrier repair process mainly via histamine H1 and H2 receptors and topical application of the histamine H1 and H2 receptor antagonists attenuated the barrier disruption-induced epidermal hyperplasia [25]. Most of the allergic symptoms stimulate further histamine release from leukocytes, consequently producing a vicious circle of disease exacerbation. Among these cells, since mast cells are the dominant source of stored histamine, the regulation of histamine release from mast cells is critical for reducing allergic symptoms and a vicious cycle of immune dysfunction.

There is accumulating evidence that keratinocytes directly take part in skin immune diseases although they are not professional immune cells: Keratinocytes are capable of responding to a variety of cytokines because of a broad range of surface receptors that they carry [26]. Activated keratinocytes are then able to produce numerous inflammatory regulators (e.g., IL-8) again that could control their own function, influence neighboring cells, or even extend the circulation and affect other sites [26]. In addition, keratinocytes are the major sources of various chemokines (e.g., MDC and TARC). Chemokines represent small, secreted molecules that regulate directional migration and importantly, mediate leukocyte trafficking [27,28]. Indeed, keratinocytes from skin allergy patients show an intrinsically unregulated chemokine level that can promote the initial immune cell recruitments into the skin, causing the commencement and maintenance of allergic symptoms [27,28]. In the current experiment, undecane treatment improved abnormally upregulated cytokine and chemokine in stimulated HaCaT cells.

In the present study, the intracellular cAMP levels increased within a few minutes after the initial exposure to undecane and returned to near basal level by 120–240 min in both mast cells and keratinocytes. Overall, similar patterns were also observed in previous studies: cAMP levels after the addition of various cAMP-elevating agents such as forskolin and prostaglandin E reached a peak mostly within 60 min in many cell types [29,30,31]. It is widely accepted that cellular cAMP levels are dynamically regulated by two classes of enzymes that act in opposition: Adenylyl cyclases (ACs) immediately stimulate cAMP signaling in response to transmembrane G protein-coupled receptor activation by an extracellular ligand, while phosphodiesterases (PDEs) perform the sole role of degradation of cAMP. An increase in cellular cAMP by ACs stimulates PKA, which directly performs anti-inflammatory activities [32]. On the other hand, there is abundant biochemical evidence that stimulated PKA activated PDEs in a variety of cells, contributing to the maintenance of restricted cAMP signals. This rapid negative feedback allows specific cellular cAMP concentrations to be achieved only for a short time, which may result in agent-specific effects [33,34,35].

Over the past several decades, downregulation of certain cytokines/chemokines in several immune cell types was considered to be a common approach to AD treatment and, indeed, the molecules targeting specific immunomodulatory pathways such as interleukin-2 (IL-2) and TNF-α demonstrated the effectiveness of this approach [36,37,38]. However, side effects have been associated with their long-term use [39]. For example, tacrolimus, the agent acting directly on T helper type 1 (Th1) to inhibit IL-2 transcription, results in Th1/Th2 imbalance with increased circulating IgE and Th2-related cytokine levels seen in vivo and in clinical trials [40,41]. It has been acknowledged that AD is immunologically complicated, relate to diverse cell types and inflammatory signals in its initiation, progression and pathological status and, therefore, a better strategy in the treatment of AD is moving from the development of molecules that are capable of mediating the physiological function of a single target to multi-target-directed ligands in a gradual way [42,43,44]. Notably, due to multiple actions of cAMP in cell physiology, cAMP has wide regulatory effects on diverse AD-related cells, including mast cells, keratinocytes, eosinophils, neutrophils and macrophages [8,45]. Further study is needed to determine the anti-inflammatory activities of undecane in other immune cells.

Although CREB activation often promotes anti-inflammatory immune responses, we unexpectedly found that treatment with inflammation-inducing agents (TNF-α/IFN-γ) increased CREB phosphorylation at Ser-133 in keratinocytes. We also investigated that the inflammatory effect of TNF-α/IFN-γ on keratinocyte contrasts with the evident anti-inflammatory effect of undecane, possibly meaning that only CREB phosphorylation is not sufficient for its anti-inflammatory effect, which further relies on the other factors. This speculation is suggested by the investigation of Montminy et al., showing stimulus-specific CREB activity. It was demonstrated that both PKA and other stimuli phosphorylated CREB at Ser-133 to similar levels. Nevertheless, only the activity of PKA led to CREB-induced anti-inflammatory activity (e.g., IL-10 gene increase) [46,47]. Thus, it was suggested that not only phosphorylation of CREB, but a second PKA-regulated stimulus is needed for transcriptional CREB activation [47]. We speculate that the contradictory results of CREB Ser-133 phosphorylation by TNF-α/IFN-γ, which did not increase the PKA levels, may be similarly understood.

**Figure 5:**
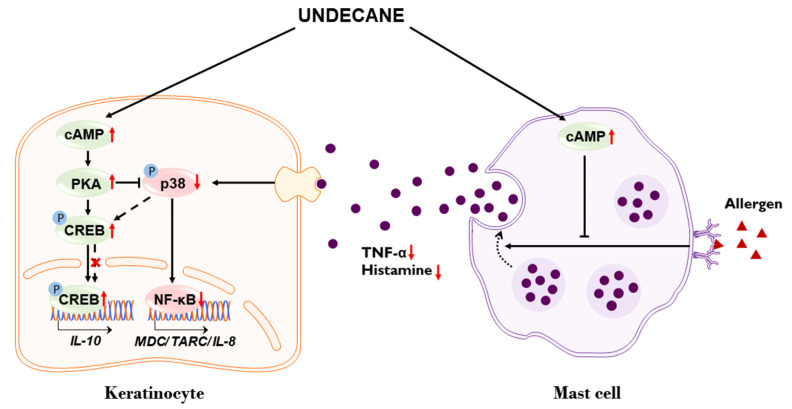
Proposed mechanism by which undecane suppresses inflammatory responses in keratinocytes and mast cells.

In summary, we demonstrated that undecane increased intracellular cAMP levels and led to the inhibition of the degranulation of mast cells and the cytokines/chemokines expression in keratinocytes (Figure 5). Based on its effects on the two cell types, which are closely responsible for the skin inflammatory processes, our data suggest that undecane may be useful for the prevention or treatment of AD-like skin inflammatory conditions and other allergic diseases. More studies are needed to further confirm the potential for developing undecane as a pharmacological strategy for ameliorating skin inflammatory diseases such as AD.

## 4. Materials and Methods

### 4.1. Cell Culture

RBL-2H3 mast cells were obtained from the American Type Culture Collection (Manassas, VA, USA) and HaCaT keratinocytes were purchased from AddexBio technologies (San Diego, CA, USA). RBL-2H3 and HaCaT cells were cultured in high-glucose Dulbecco’s modified Eagle’s medium (DMEM; HyClone, Logan, UT, USA) supplemented with 10% heat-inactivated fetal bovine serum (FBS; 56 °C for 30 min; Gibco, Grand Island, NY, USA) and 1% 100 unit/mL penicillin–streptomycin (Gibco, Grand Island, NY, USA). The cells were grown at 37 °C in a 5% CO_2_ atmosphere incubator (Sanyo, Osaka, Japan).

### 4.2. Cell Viability Assay

Cell viability was determined by MTT (Sigma Aldrich, St. Louis, MO, USA) assay. RBL-2H3 (1 × 10^4^ cells/well) and HaCaT cells (1 × 10^4^ cells/well) were seeded in 96-well plates and incubated for 24 h. The cells were treated with or without 1–100 μM of undecane (purity ≥99%; Sigma Aldrich, St. Louis, MO, USA) and cultured for an additional 24 h. After the media was discarded, MTT solution (4 mg/mL in phosphate-buffered saline (PBS; Welgene, Daegu, Korea)) was added to each well and the cells were incubated for further 3 h. Thereafter, the supernatant was aspirated and the formazan crystals were solubilized in dimethyl sulfoxide (DMSO; Sigma Aldrich, St. Louis, MO, USA) for 30 min. Relative absorbance was measured at 570 nm with an Infinite M200 PRO microplate reader (Tecan, Männedorf, Switzerland).

### 4.3. Toluidine Blue Staining

RBL-2H3 cells were seeded into 24-well plates at 6 × 10^4^ cells/well and incubated for 24 h. The cells were then cultured for 1 h in serum-free medium containing vehicle (DMSO) or undecane (0.5, 1 and 5 μM), followed by sensitization with 50 ng/mL of DNP-IgE (Sigma Aldrich, St. Louis, MO, USA) for 4 h. The sensitized cells were washed two times with PBS and degranulation was induced by 50 ng/mL of DNP-HSA (Sigma Aldrich, St. Louis, MO, USA). After 1 h, the cells were washed again with cold PBS and fixed in 4% paraformaldehyde (Nest Biotechnology, Rahway, NJ, USA) at 25 °C. In the next step, 0.1% toluidine blue (Sigma Aldrich, St. Louis, MO, USA) in 1% sodium chloride (pH 2.5) was added to each well and cells were incubated for 30 min. After that, the stained cells were washed three times with PBS. Images of the stained cells were captured with an Olympus IX71 microscope using a DP-70 controller (Olympus, Center Valley, PA, USA).

### 4.4. cAMP Assay

RBL-2H3 and HaCaT cells were seeded into 24-well plates at a density of 6 × 10^4^ cells/well. RBL-2H3 and HaCaT cells were incubated with 1 and 5 μM undecane, respectively, for 15, 30, 60, 120 and 240 min or without undecane treatment. At the end of incubation, the medium was aspirated and the cells were lysed with 0.1 M HCl for 10 min and scraped off and the cell lysates were collected. cAMP concentrations of the cell lysates were measured with the cAMP ELISA kit (Enzo, Plymouth Meeting, PA, USA) following the manufacturer’s protocols. The relative absorbance was measured at 400 nm using a microplate reader (Tecan, Männedorf, Switzerland). The cAMP level was normalized to the total intracellular protein amount. The protein amount in cell lysates was estimated using the Bradford reagent (Bio-Rad, Hercules, CA, USA). Bovine serum albumin (BSA; LPS solution, Daejeon, Korea) was used as the standard.

### 4.5. Histamine and TNF-α Release Assays

RBL-2H3 cells were seeded onto a 12-well plate at 1 × 10^5^ cells/well, cultured and sensitized using the above-mentioned methods for toluidine blue staining. After induction of degranulation, the supernatants were centrifuged (12,000× *g*, 5 min, 4 °C) and collected. Histamine and TNF-α concentrations in the supernatants were estimated using ELISA kits (Enzo, Plymouth Meeting, PA, USA) according to the manufacturer’s instructions. The histamine and TNF-α concentrations were normalized to the total cellular protein amount. The total cellular proteins were extracted with cell lysis buffer containing 100 mmol/L Tris–HCl (pH 7.4), 100 mmol/L orthovanadate, 50 mmol/L sodium pyrophosphate, 50 mmol/L NaF, 50 mmol/L NaCl, 5 mmol/L EDTA, 1 mmol/L phenylmethanesulfonyl fluoride, 1% Triton X-100, 2 μg/mL aprotinin, 1 μg/mL pepstatin A and 1 μg/mL leupeptin. The protein amount was evaluated with the Bradford reagent as described above.

### 4.6. NF-κB p65 Transcriptional Activity

HaCaT cells were seeded into 60-mm cell culture dishes at 3 × 10^5^ cells/well and incubated for 24 h. The cells were then cultured for 1 h in serum-free medium containing vehicle or various concentrations of undecane (1, 5 and 10 μM), followed by sensitization with 10 ng/mL each of TNF-α (R&D Systems, Minneapolis, MN, USA) and IFN-γ (R&D Systems, Minneapolis, MN, USA) for 1 h. Nuclear proteins were then separated from HaCaT cells using the Nuclear Extraction Kit (Abcam, Cambridge, MA, USA) and NF-κB (a heterodimer of p50 and p65 subunits) activation was assessed using NF-κB p65 transcription factor assay kit (Abcam, Cambridge, MA, USA) following the manufacturer’s protocol. Absorbance was quantified with a microplate reader (Tecan, Männedorf, Switzerland) at 450 nm.

### 4.7. Western Blotting

HaCaT cells were seeded into 60-mm cell culture dishes at 3 × 10^5^ cells, cultured for 24 h and sensitized with TNF-α/IFN-γ (10 ng/mL each) with or without undecane (5 μM). The cells were then harvested with cell lysis buffer, incubated for 10 min and centrifuged at 13,000× *g* for 5 min at 4 °C. The concentrations of proteins were determined by Bradford assay as described above. The prepared samples containing 20 µg total protein were separated in 10–12% SDS-PAGE gel and transferred onto nitrocellulose membranes (Whatman, Dassel, Germany). After transfer, the membranes were blocked with 5% BSA in TBST (20 mmol Tris–HCl, pH 7.6, containing 137 mmol NaCl and 0.05% Tween-20). Primary antibody incubation was performed overnight at 4 °C in the presence of PKA (Cat# 4782), glyceraldehyde-3-phosphate dehydrogenase (GAPDH; Cat# 5174), CREB (Cat# 9197), p-CREB (Cat# 9198), p38 (Cat# 8690) and p-p38 (Cat# 4511) (Cell Signaling, Danvers, MA, USA) using a 1:1000 dilution for each antibody. Membranes were then incubated with secondary antibody (Cat# sc-2055; 1:10,000 dilution; Santa Cruz Biotechnology, Santa Cruz, CA, USA) for 1 h at 20 °C. Electrochemiluminescence (ECL) detection reagent (Bio-Rad, Hercules, CA, USA) was used as a substrate and the band images were captured with a WSE-6100 LuminoGraph system (ATTO, Tokyo, Japan). Band intensities were calculated with the Quantity One software (v 4.6.2, Bio-Rad, Hercules, CA, USA) and normalized to the loading control (GAPDH).

### 4.8. Reverse Transcription and Quantitative Real-Time Polymerase Chain Reaction

HaCaT cells were seeded into 6-well plates at 3 × 10^5^ cells/well and incubated for 24 h. The cells were then cultured for 1 h in serum-free medium containing vehicle or undecane (5 μM), followed by sensitization with 10 ng/mL each of TNF-α and IFN-γ for 6 h. Then, the treated cells were harvested and RNA was isolated using the Trizol (Invitrogen, Carlsbad, CA, USA) following the manufacturer’s protocol. RNA was reverse transcribed to generate cDNA according to the SuperScript IV reverse transcriptase (Invitrogen, Carlsbad, CA, USA) protocol: 2 μg of RNA was reverse transcribed in the 5× SuperScript IV buffer supplemented with 50 μM oligo (dT) primer, 200-unit SuperScript IV reverse transcriptase, 10 mmol dNTP mixture and 20 unit RNase inhibitor. The reaction was incubated at 52 °C for 1 h. Real-time polymerase chain reaction (PCR) quantification was conducted with a real-time PCR system (CFX96 Touch; Bio-Rad, Hercules, CA, USA) in a 20 μL mixture containing 10 μL of SYBR Green fluorescence (iQ SYBR Green Supermix; Bio-Rad, Hercules, CA, USA), 20 pmol primers and 50 ng of cDNA template. The relative gene levels were estimated by the threshold cycle method, using GAPDH as the housekeeping gene. Primer sets used in quantitative real-time PCR are given in Table 1.

## Figures and Tables

**Table 1:** Primer sequences.

Gene Description	Sequence (5′→3)
Thymus and activation-regulated chemokine (TARC)	F: CCTGGTCACCCTCCTCCTG R: GGTACCACGTCTTCAGCTTTCT
Macrophage-derived chemokine (MDC)	F: CCCTACGGCGCCAACAT R: CAGACGGTAACGGACGTAATCA
Interleukin-8 *(*IL-8*)*	F: TGACTTCCAAGCTGGCCGTG R: TTCTGTGTTGGCGCAGTGTGG
IL-10	F: GATCCAGTTTTACCTGGAGGAG R: CCTGAGGGTCTTCAGGTTCTC
Glyceraldehyde-3-phosphate dehydrogenase (GAPDH)	F: GTGATGGCATGGACTGTGGT R: GGAGCCAAAAGGGTCATCAT
