# Correction: Choi et al. Anti-Allergic and Anti-Inflammatory Effects of Undecane on Mast Cells and Keratinocytes. *Molecules* 2020, *25*, 1554

**DOI:** 10.3390/molecules30081768

**Published:** 2025-04-15

**Authors:** Dabin Choi, Wesuk Kang, Taesun Park

**Affiliations:** Department of Food and Nutrition, Brain Korea 21 PLUS Project, Yonsei University, 50 Yonsei-ro, Seodaemun-gu, Seoul 03722, Korea

The authors would like to modify the Conflicts of Interest Section of the following published paper [1]. The changes are below.

**Conflicts of Interest:** The authors would like to specify that the corresponding author, Taesun Park, holds several patents and is the CEO of a cosmetic company that commercializes products that contain similar ingredients to those that are being investigated in the abovementioned article. The company, BOTANICSENS Inc., had no involvement in the design of the study, the collection, analysis, or interpretation of the data, the writing of the manuscript, or the decision to publish the result.

The authors apologize for any inconvenience caused and state that the scientific conclusions are unaffected.

This correction was approved by the Academic Editor. The original publication has also been updated. 
